# Potential diagnostic application of a novel deep learning- based approach for COVID-19

**DOI:** 10.1038/s41598-023-50742-9

**Published:** 2024-01-02

**Authors:** Alireza Sadeghi, Mahdieh Sadeghi, Ali Sharifpour, Mahdi Fakhar, Zakaria Zakariaei, Mohammadreza Sadeghi, Mojtaba Rokni, Atousa Zakariaei, Elham Sadat Banimostafavi, Farshid Hajati

**Affiliations:** 1 Intelligent Mobile Robot Lab (IMRL), Department of Mechatronics Engineering, Faculty of New Sciences and Technologies, University of Tehran, Tehran, Iran; 2 Student Research Committee, Mazandaran University of Medical Sciences, Sari, Iran; 3 Pulmonary and Critical Care Division, Imam Khomeini Hospital, Mazandaran University of Medical Sciences, Sari, Iran; 4 Iranian National Registry Center for Lophomoniasis and Toxoplasmosis, Imam Khomeini Hospital, Mazandaran University of Medical Sciences, P.O Box: 48166-33131, Sari, Iran; 5 Toxicology and Forensic Medicine Division, Mazandaran Registry Center for Opioids Poisoning, Anti-microbial Resistance Research Center, Imam Khomeini Hospital, Mazandaran University of Medical Sciences, P.O box: 48166-33131, Sari, Iran; 6 Department of Radiology, Qaemshahr Razi Hospital, Mazandaran University of Medical Sciences, Sari, Iran; 7 MSC in Civil Engineering, European University of Lefke, Nicosia, Cyprus; 8 Department of Radiology, Imam Khomeini Hospital, Mazandaran University of Medical Sciences, Sari, Iran; 9 Intelligent Technology Innovation Lab (ITIL) Group, Institute for Sustainable Industries and Liveable Cities, Victoria University, Footscray, Australia

**Keywords:** Biotechnology, Computational biology and bioinformatics, Developmental biology, Engineering

## Abstract

COVID-19 is a highly communicable respiratory illness caused by the novel coronavirus SARS-CoV-2, which has had a significant impact on global public health and the economy. Detecting COVID-19 patients during a pandemic with limited medical facilities can be challenging, resulting in errors and further complications. Therefore, this study aims to develop deep learning models to facilitate automated diagnosis of COVID-19 from CT scan records of patients. The study also introduced COVID-MAH-CT, a new dataset that contains 4442 CT scan images from 133 COVID-19 patients, as well as 133 CT scan 3D volumes. We proposed and evaluated six different transfer learning models for slide-level analysis that are responsible for detecting COVID-19 in multi-slice spiral CT. Additionally, multi-head attention squeeze and excitation residual (MASERes) neural network, a novel 3D deep model was developed for patient-level analysis, which analyzes all the CT slides of a given patient as a whole and can accurately diagnose COVID-19. The codes and dataset developed in this study are available at https://github.com/alrzsdgh/COVID. The proposed transfer learning models for slide-level analysis were able to detect COVID-19 CT slides with an accuracy of more than 99%, while MASERes was able to detect COVID-19 patients from 3D CT volumes with an accuracy of 100%. These achievements demonstrate that the proposed models in this study can be useful for automatically detecting COVID-19 in both slide-level and patient-level from patients’ CT scan records, and can be applied for real-world utilization, particularly in diagnosing COVID-19 cases in areas with limited medical facilities.

## Introduction

Although over the past two decades, viruses from the coronavirus family and influenza virus have been some of the most prevalent pathogens causing respiratory illnesses^1^, in late 2019, the SARS COV-2 virus caused the COVID-19 disease, which on March 11, 2020, was declared as a global pandemic by the World Health Organization (WHO)^2^. The clinical manifestations of COVID-19 are very diverse, patients can be asymptomatic or have mild symptoms such as fatigue, fever, body aches, sore throat, headache, diarrhea, dry cough, and shortness of breath to severe and life-threatening manifestations such as chest pain, confusion, decreased level of consciousness and pneumonia, which requires hospitalization in the intensive care unit and the use of mechanical ventilation^3^. Early detection of this infection in the patient's body is an important and essential step to prevent the coronavirus's spread. Currently, the gold standard for diagnosing the disease of COVID-19 is the reverse transcription polymerase chain reaction, which, among its disadvantages, is its low sensitivity (60–71%)^4–6^ On the other hand, the analyzes showed that about 84% of the patients with COVID-19 have abnormal findings in computed tomography (CT scan) and chest x-ray (*p* < 0.001)^7^, in which the sensitivity of CT scan for its diagnosis is also up to 97%^4^, therefore, a CT scan of the chest plays a very big role in identifying pneumonia caused by COVID-19, and some studies indicate that a CT scan can be used as the first auxiliary tool for screening and identifying patients in pandemic areas^8–10^.

The most common radiological manifestations in COVID-19-positive patients are bilateral involvement in chest X-ray, opacities, and ground-glass opacities^7^, but it should be noted that radiological findings in pneumonia caused by COVID-19 are not specific, and can overlap with other viral pneumonia, so identifying the differences between the radiological findings in this disease and other viral pneumonia in differentiating them is the main challenge for screening these patients and helping to break the chain of disease transmission. In pandemic areas, the number of patients visiting medical centers is very high, so physicians cannot review CT scan images in a short time and this process is prolonged. This issue can lead to an increase in the risk of infection in some patients and make the treatment process difficult for them. In addition, due to work pressures and the inevitability of human error, the possibility of errors in the diagnosis of these images by the physicians is very likely.

Artificial intelligence (AI) refers to the use of computer systems to model intelligent behaviors without human intervention^11^. It encompasses several subfields, including machine learning, which focuses on systems learning from existing examples^12^. However, machine learning has limitations, such as the inability to identify complex nonlinear relationships and the need for an expert to interpret data for the system^13^. Recent hardware advancements led to the development of deep learning, a subfield of AI that uses larger models with more layers to learn complex data. Deep learning systems can identify many complex nonlinear relationships and learn the final output without human intervention^14^. This has led to remarkable performances in complex data analysis, such as image processing^15^, where deep learning systems have even surpassed human performance.

Deep learning systems can be particularly useful in the diagnosis of diseases, such as diagnosing malaria from blood smear images^16–18^. These systems can diagnose patients with high accuracy in a short time, and assist medical staff in diagnosing the disease more accurately and quickly, especially in cases of pandemics or endemic diseases such as COVID-19. The initial research on deep learning techniques for detecting COVID-19 from CT scans lacked transparency in terms of publishing the dataset on which their models were trained^19,20^. Moreover, in the investigation in this field, most studies have focused on slide-level analysis which means deep learning models were specifically designed for the classification of individual CT scan slides rather than the patients themselves^21–25^. Other studies have developed deep learning models for patient-level analysis^26–29^. These studies also benefit from 2D models to analyze each slice of a patient's CT scan separately and then use a specific decision process to make a final prediction about the patient's condition based on these slice analyses.

The quality and quantity of CT scan datasets utilized in training deep learning models for the detection of COVID-19 represent pivotal considerations^14^. Suboptimal CT scan images, characterized by factors such as poor clarity or extraneous content, may compromise the system's accuracy in diagnosing COVID-19. Furthermore, a limited number of CT scan images within the dataset could impede the system's performance when faced with new patients, potentially rendering it unreliable. Therefore, the establishment of a reliable deep learning model necessitates the incorporation of a large and diverse dataset encompassing various manifestations of COVID-19 in CT scans.

Our study's contributions and main novelties are as follows:

- Firstly, we introduce a novel dataset, COVID-MAH-CT, containing chest CT images belong to COVID-19 patients. These CT scan images are selected from patients from four different periods related to the four different waves of the COVID-19 epidemic in Iran. This selection can provide better and more reliable training for more accurate diagnosis by considering the diversity of pulmonary involvement patterns in different waves. COVID-MAH-CT can be a resource for future research in the development of deep learning models for detecting diseases related to the lungs for both slide-level and patient-level. To illustrate the dataset's practicality, we conducted a comprehensive evaluation by applying various transfer learning models to assess its potential for slide-level analysis.
- Another contribution of this study is to train a novel 3D deep learning model to detect COVID-19 patients from 3D CT scan images. In many previous studies, the approach was to analyze individual slices of CT scans to determine COVID-19 status, a method that requires accurate labeling of each slice within a scan. However, this can be quite impractical in real-world situations. Our model, the Multi-Head Attention Squeeze and Excitation Residual Network (MASERes network), stands out because it implements 3D convolutional layers and as a result, is able to work with 3D CT scans as a whole, and it doesn't require detailed labeling of every single slice. This makes it a significant advancement in situations where only one label for an entire set of CT scans is available, which is often the case in clinical practice.

The paper is organized as follows: In the Materials and Methods section, we extensively detail our novel dataset, COVID-MAH-CT, and explain the techniques utilized for slide-level and patient-level analyses, along with insights into the MASERes network's structure. The Results section reports the outcomes of both slide-level and patient-level analyses, includes findings from applying MASERes to a COVID-19 dataset other than COVID-MAH-CT, and presents the results of an ablation study on MASERes. In the Discussion section, we comprehensively review other studies related to patient-level COVID-19 detection through CT scans, drawing comparisons and identifying their respective limitations and advantages. Lastly, the Conclusion section wraps up the paper.

## Materials and methods

### COVID-MAH-CT

The COVID-MAH-CT dataset comprises chest CT scans of 133 patients who tested positive for COVID-19 and were referred to training-therapeutic hospitals in northern Iran from March 20, 2020, to March 13, 2022. The patients were selected purposefully during four different waves of the COVID-19 epidemic in Iran, which distinguishes this study from comparable studies conducted worldwide.

In this study, a patient is considered positive for COVID-19 infection if they meet one or more of the following criteria:

1. Suspicious clinical symptoms (including fatigue, fever, body aches, sore throat, headache, diarrhea, dry cough, etc.) along with positive radiological findings in chest CT scan
2. Suspicious laboratory findings (leukopenia and lymphopenia, impaired erythrocyte sedimentation rate markers, and C-reactive protein) along with positive radiological findings in chest CT scan
3. (COVID-19) positive RT-PCR with positive radiological findings in chest CT scan

The CT scan images used for the diagnosis and treatment of patients in training-therapeutic centers were captured by CT scan imaging devices of the same model, SOMATOM Scope-Siemens-Germany, and visualized using the same software version, syngo CT VC30-easyIQ. The exported radiology images were in 16-bit DICOM format with a resolution of 512 × 512 pixels. To ensure patient privacy and facilitate the use of standard programming libraries, we converted the images to TIFF format, which retains the 16-bit data. Three radiologists examined the images and classified them as positive or negative for COVID-19. Positive radiological findings are also in the form of (1) Ground glass opacities (GGOs) (foggy transparent opacities), (2) Crazy pavement (thickening of interlobular septa and intralobular lines on a background of opaque glass) and (3) Consolidation pattern (meaning the air in the peripheral alveoli and bronchioles has been replaced by liquid). In cases where the diagnosis of one radiologist differed from the diagnoses of the other two experts, we considered the more probable diagnosis based on the diagnoses of the two other experts. We excluded images that showed visible lesions related to other diseases or known masses, images with unfavorable quality, and those with insufficient radiation from the study.

To enhance the accessibility and usability of the COVID-MAH-CT, we have created two separate versions of the dataset. Specifically, we have compiled a dataset called 3D-COVID-MAH-CT, which contains chest CT volumes from 133 patients who have tested positive for COVID-19. Additionally, our team of radiologists carefully examined all CT scan records of these patients and selected only those slices that exhibited signs of pulmonary infection. As a result, we have gathered a total of 4442 CT slices, which are included in the 2D-COVID-MAH-CT dataset. Access to both databases is available via the web link provided in the data availability section located at the end of the paper. In addition, clinical data for patients including age, gender, date, treatment center, Peak Kilo voltage (kV), X-ray Tube Current (mA), and Slice Thickness (mm) are also collected and made available to other researchers.

### Slide-level analysis

**Figure 1:**
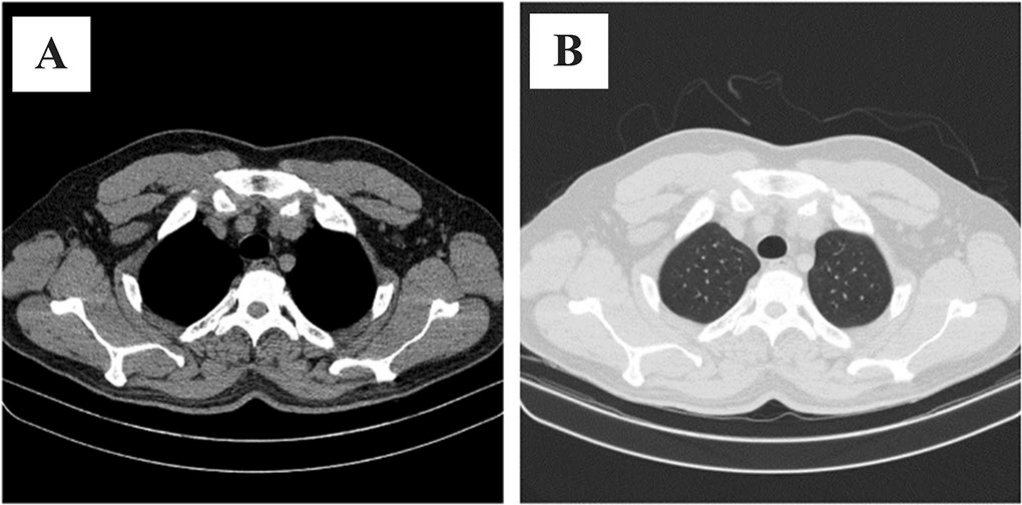
(**A**) a sample obtained from^30^ in its original abdomen view. (**B**) the same sample transformed into the lung view.

In this section, we propose several models that benefit from the transfer learning technique to detect COVID-19 in CT scan slides. Although the 2D-COVID-MAH-CT dataset exclusively contains COVID-19 samples, in order to effectively train the models for the classification task, it is necessary to incorporate normal CT scans. In order to achieve this, we incorporated normal images that were obtained from^30^, which were taken from Iranian patients and therefore have the same distribution as our dataset. Specifically, we selected normal images from the first 50 patients in this dataset, resulting in a collection of 5216 normal images, all of which had a resolution of 512 × 512 (matching our dataset). However, the images in^30^ were provided with an abdomen view and therefore required transformation to a lung view before being utilized for the purpose of this study as demonstrated in Fig. 1A,B.

In the rest of this section, the preprocessing steps applied to the CT slides are presented first, followed by a detailed discussion of the architecture of the proposed models.

#### CT slides preprocessing

The total CT scan slides were divided into three distinct sets: a training set, a validation set, and a test set. The training set was used to train the proposed models, while the validation and test sets were used for validation and testing purposes, respectively. Out of the CT slides in the dataset, 7726 (80%) were randomly assigned to the training set, while the validation and test sets each contained 966 samples (10%). It is worth noting that the ratio of COVID-19 positive samples to normal samples was maintained approximately equal across all three sets to ensure a reliable evaluation of the models. As a result, the training set comprised of 3554 COVID-19 and 4172 normal samples, while each of the validation and test sets contained 444 COVID-19 and 522 normal slides. Detailed information of the data split performed in our study for the slide-level analysis is provided in Table 1.

**Table 1:** Details of data split in slide-level analysis.

	Number of slides		
	Total	COVID-19	Normal
Training set	7726 (80%)	3554	4172
Validation set	966 (10%)	444	522
Test set	966 (10%)	444	522
Total slides	9658	4442	5216

For the purpose of preprocessing in slide-level analysis, a series of transformations were applied to the input images as part of a standard procedure to enhance their suitability for training deep neural networks. Initially, we rescaled the pixel values of the input images, ensuring that they fell within the normalized range of 0–1. This normalization practice facilitates the training of deep neural networks by ensuring that the input data falls within a consistent and manageable range.

Additionally, we adjusted the dimensions of the input images to align them with the requirements of different deep neural network models employed in our study, taking into consideration variations in receptor sizes among these models. Consequently, the dimensions of the input images were adjusted to 224 × 224 pixels for VGG19, ResNet50V2, DenseNet169, and MobileNetV2, while InceptionV3 and Xception were configured with input images of 229 × 229 pixels.

In addition to these preprocessing steps, we introduced data augmentation techniques to enhance the robustness of the models. Firstly, random rotations ranging from − 40° to + 40° were applied to the input images to increase the models' invariance to rotation. Furthermore, horizontal and vertical shifts of up to 20% of the image's width or height were randomly introduced to simulate changes in perspective. Shear intensity up to 0.2 was applied to create a slanting effect, and random zooming in or out by up to 20% was employed to encourage the model to recognize objects at different scales. Horizontal flipping was also incorporated to randomly flip images horizontally. To address changes in image size or shape resulting from these transformations, a nearest-neighbor strategy was employed to fill in newly created pixels, simply replicating the nearest pixel value to bridge the gaps.

It is crucial to emphasize that all the data augmentation techniques mentioned above were exclusively utilized during the training phase and were not applied to the validation and testing datasets. For these validation and testing phase, the only preprocessing steps performed were the rescaling of image pixels to the range between 0 and 1 and the adjustment of the input image size to the appropriate dimensions required by the models. This differentiation ensures the integrity of the validation and testing processes by maintaining a consistent evaluation environment.

#### Slide-level models’ architecture

The performance of deep learning models is heavily dependent on the quality and quantity of the dataset used for training^31^. A larger and cleaner dataset is likely to result in a better training process and, consequently, a better performing model. However, in healthcare-related studies, obtaining a suitable dataset is often challenging due to ethical and practical considerations, resulting in small-sized medical databases^32–34^. As a result, training deep models from scratch for medical purposes is difficult. To address this challenge, the transfer learning method is a crucial technique. The idea of the transfer learning is to use a pre-trained model that has already learned features from a large dataset, and then fine-tune it on a smaller dataset specific to the new task^35^. Transfer learning has been widely used in computer vision^36^ and natural language processing^37^.

We trained six convolutional neural networks using the transfer learning method and evaluated their performance on the 2D-COVID-MAH-CT dataset for detecting COVID-19 slides. The feature extraction part of these models were retrieved from various pre-trained models, including VGG19^38^, InceptionV3^39^, ResNet50V2^40^, Xception^41^, MobileNetV2^42^, and DenseNet169^43^. In the following paragraphs, we provide brief information about each of these pre-trained models used in our study.

##### VGG19

Simonyan and Zisserman^38^, proposed by the Visual Geometry Group at the University of Oxford, can be regarded as one of the pioneering models that benefits from expanding model's depth to achieve higher accuracy. This model, which comprises convolutional, pooling, and dense layers achieved a high ranking in the ImageNet Large Scale Visual Recognition Competition (ILSVRC)^44^ in 2014, underscoring its effectiveness in image classification tasks.

##### InceptionV3

Szegedy et al.^39^is an extension of the Inception family of models, which are built to improve accuracy and efficiency by using Inception modules^45^. These modules allow the network to simultaneously apply multiple filters to each input layer, improving its ability to detect complex patterns in images.

##### ResNet50V2

He et al.^40^ is an enhanced version of the original ResNet50^46^ model that utilizes residual blocks. These blocks enable the training of very deep networks by addressing the degradation issue and accuracy saturation problem that can arise with traditional deep neural network architectures. Additionally, ResNet50V2 incorporates several other improvements over the original ResNet50 model, including enhanced batch normalization and a revised bottleneck architecture.

##### Xception

Chollet^41^ (Extreme Inception) is an extension of the Inception architecture that also benefits from skip connectors. Moreover, Xception replaces the standard convolutional layers in Inception with Depthwise separable convolutions^47^. This reduces the number of parameters and computations required in the model, leading to faster training and improved accuracy.

##### MobileNetV2

Sandler et al.^42^ is an upgraded version of MobileNet^48^, optimized for mobile and embedded vision applications. This model is fast and accurate enough for real-world applications thanks to utilizing Depthwise Separable Convolutions^47^.

##### DenseNet169

Huang et al.^43^ is part of the DenseNet family of models and is based on the idea of densely connected layers, where each layer receives inputs from all its preceding layers. This configuration ensures that the input of a particular layer in DenseNet is a concatenation of all outputs of the previous layers. By adopting this architecture, DenseNet has made considerable advancements in computer vision tasks, offering a solution to the gradient vanishing problem that previously plagued deep convolutional models.

For the purpose of this study, a binary classification model is required while all the mentioned pre-trained models have been originally designed for multiclass classification. Consequently, the dense part of each pre-trained model, which is responsible for multiclass classification, was eliminated and replaced with a new dense part optimized for the binary classification. In our new classification module, there exist two distinct layers where the first one comprises four individual neurons, and the second layer is constituted by a single neuron that uses a sigmoid activation function. In order to mitigate the potential effects of overfitting, Dropout layer^49,50^ with a drop ratio of 0.25 has been incorporated into the first layer.

### Patient-level analysis

Due to the fact that the manifestations of COVID-19 may not be present in all CT scan slides of a given patient, relying solely on slide-level analysis may lead to inefficiencies and potential misdiagnosis. This can ultimately compromise the treatment process and quarantine policies. Therefore, it is crucial to conduct patient-level analysis, which takes into account the entire set of a patient's CT scan slides when evaluating COVID-19 patients.

To address this issue, we developed the Multi-Head Attention Squeeze and Excitation Residual network (MASERes network) and evaluated its performance on the 3D-COVID-MAH-CT dataset to automatically detect COVID-19 patients by analyzing the entire set of CT scan slides. It should be noted that this dataset exclusively contains CT scans of patients with COVID-19, and therefore, we required a separate dataset consisting of CT scans from normal patients to facilitate the classification task. To this end, we utilized the normal CT scans from^30^ which were obtained from 76 patients and were originally captured in abdomen view. In order to align these scans with our research objectives, we transformed them from abdomen view to lung view and exported them as TIFF images for further analysis.

#### CT volumes preprocessing

Our study involved patient-level analysis using a total of 133 COVID-19 CT volumes from 3D-COVID-MAH-CT and 76 CT volumes from COVID-CT-MD^30^. Out of these, we randomly selected 168 (80%) for the training process, and two sets of 20 and 21 CT volumes were collected for the validation and testing processes, respectively. Importantly, this split was designed such that the ratio of COVID-19 volumes to normal volumes in all three sets remained equal to ensure reliable model evaluation. As a result, the training set consisted of 107 COVID-19 CT volumes and 61 normal CT volumes, while the validation set contained 13 COVID-19 CT volumes and 7 normal CT volumes. The test set included 13 COVID-19 CT volumes and 8 normal CT volumes. Detailed information of the data split performed in our study for the slide-level analysis is provided in Table 2.

**Table 2:** Details of data split in patient-level analysis.

	Number of CT volumes		
	Total	COVID-19	Normal
Training set	168 (80.4%)	107	61
Validation set	20 (9.6%)	13	7
Test set	21 (10%)	13	8
Total CT volumes	209	133	76

**Figure 2:**
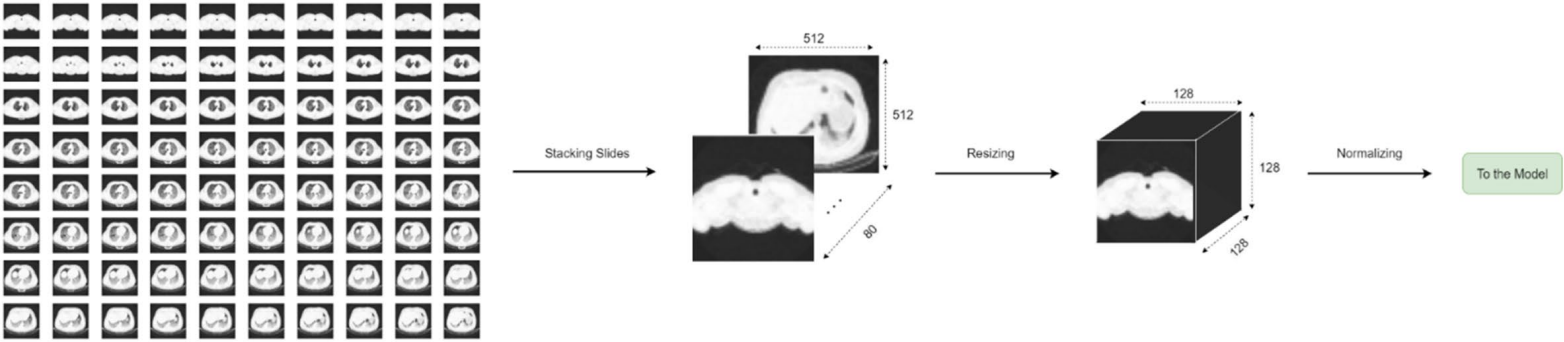
Preparing CT volumes for the patient-level analysis.

It was observed that the CT volumes of each patient contained varying numbers of slides, ranging from 50 to over 150, which encompassed the thorax inlet to thorax outlet. However, all slides had the same dimensions of (512 × 512). To standardize the input size of the samples, we first stacked all the slides of a given patient in chronological order, as depicted in the Fig. 2. Thereafter, we resized all the CT volumes from their original dimensions of (512 × 512 × different numbers) to a unified size of (128 × 128 × 128). Finally, to address the amplitude scaling and offset effect, all volumes were standardized using the z-score method^51^.

To enhance the robustness of the MASERes model when dealing with input volumes at varying angles, we implemented a data augmentation methodology. Specifically, this approach involves the application of random rotations to the input volumes. To achieve this, we introduced a predefined list of rotation angles, namely [− 20, − 10, − 5, 0, 5, 10, 20] degrees. During the augmentation process, the model randomly selects one angle from this list and applies it to the input volume. This technique contributes significantly to the model's ability to maintain robust performance when confronted with rotated CT volumes. It is essential to note that the described augmentation technique is exclusively applied to the training volumes and does not extend to the validation and testing CT volumes. In practice, the validation and testing CT volumes undergo only two preprocessing steps: resizing and standardization.

#### Multi-head attention squeeze and excitation residual neural network (MASERes)

**Figure 3:**
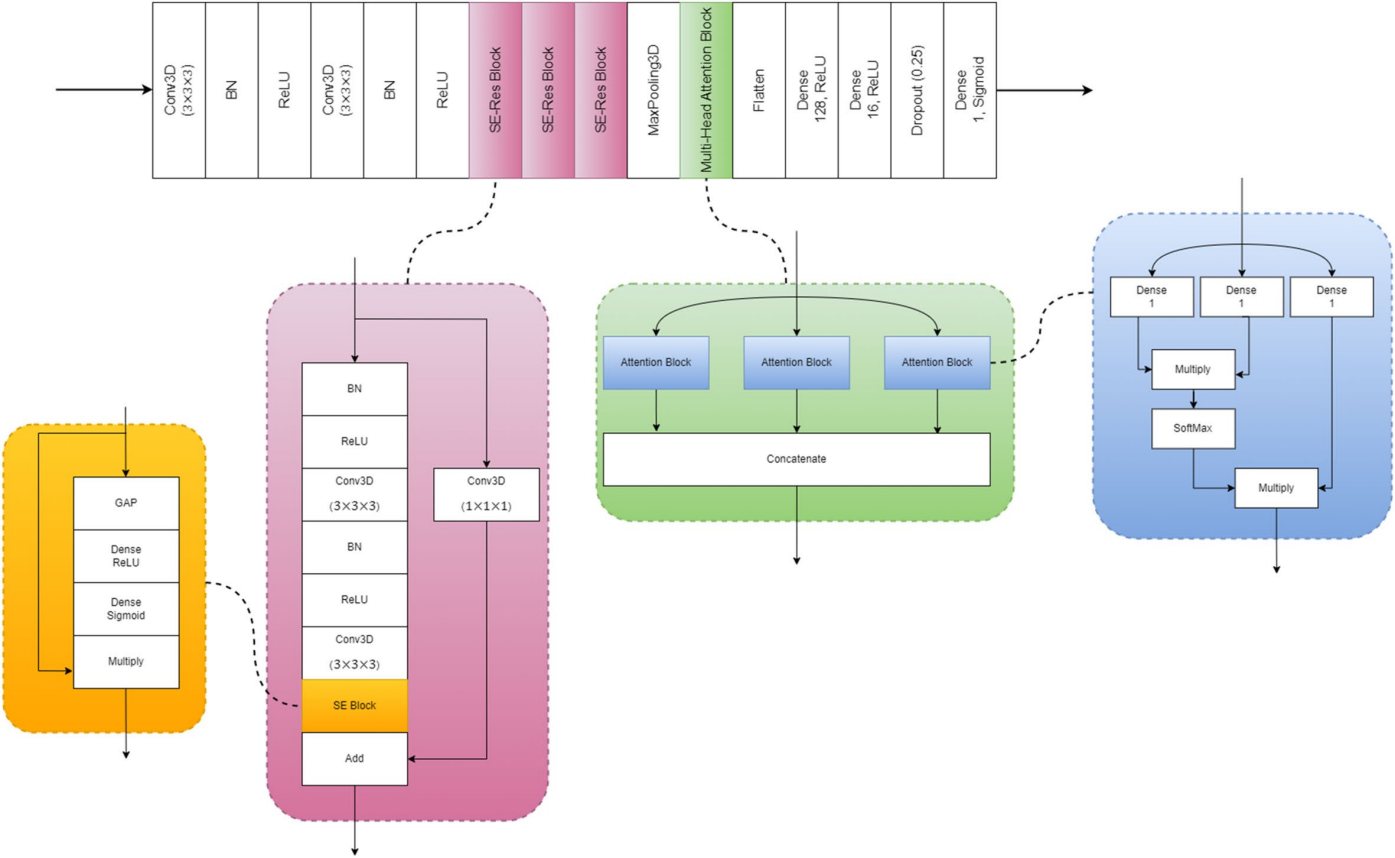
The architecture of MASERes, which includes three SE-Res blocks and a multi-head self-attention block with three self-attention mechanisms.

Figure 3 depicts the architecture of the Multi-Head Attention Squeeze and Excitation Residual Neural Network (MASERes Neural Network), which has been designed to analyze 3D CT volumes and detect patients with COVID-19. The first few layers of MASERes consist of 3D convolutional and batch normalization layers to capture the low-level features of the CT volumes. Notably, all convolutional layers implemented in MASERes have a filter size of (3 × 3 × 3) as 3 is the smallest value to capture information from a focal point and its immediate neighbors. A group of three stacked Squeeze and Excitation Residual blocks (SE-Res blocks) are then implemented to retrieve high-level features and important channels.

In an input vector, values in different channels do not have an equal impact on the final output, and therefore have varying levels of importance. As a result, it is crucial to detect the more important channels and magnify their impact on the final output. This is achieved through the use of Squeeze and Excitation (SE) blocks^52^. SE blocks utilize a global average pooling (GAP) technique first to convert the information of each channel into a scalar. These scalars are then processed through two consecutive neural networks, resulting in a vector containing the scalars that represent the importance of each channel.

1 $${X}_{SE-output}=s \times {X}_{SE-input}$$

2 $$s= \sigma ({W}_{2} \times {\delta (W}_{1 }\times \stackrel{-}{x))}$$

Where $\sigma$ and $\delta$ are sigmoid and ReLU^53^ activation functions respectively. In addition, ${W}_{1}$ and ${W}_{2}$ are defined through the training process and $\overline{x }$ is the output of the global average pooling layer.

SE blocks in MASERes are implemented in conjunction with the residual blocks which are helpful to address the gradient vanishing problem in deep neural networks^40,46^. SE-Res block can effectively capture the high-level features of inputs.

3 $${X}_{SE-Res-output}={F}_{1}\left({F}_{2}\left({X}_{SE-Res-output}\right)\right)+ {X}_{SE-Res-input}$$

where F_2_ (X) is the transformation function through the Conv1D layers in the residual block, and F_1_ (X) is the transformation function of the SE block. Upon the completion of the final SE-Res block, the output is directed to a max pooling layer which purpose is to decrease the feature size of the output. Following this, a multi-head attention^54^ part is employed which is designed to contain three distinct self-attention^55^ mechanisms.

Self-attention mechanisms are employed to intensify more important parts of a given input by generating attention weights. These weights represent the importance of each element in the input sequence relative to the others, allowing the network to selectively focus on the most relevant information. To generate attention weights, different techniques can be employed. In this study, we utilized a method similar to the one presented in reference^54^. This method is represented in Eqs. (4) and (5) and has proven to be effective in enhancing the network's performance.

4 $$ \left\{ \begin{gathered} K = W_{k}^{{}} .X_{att - input}^{{}} \hfill \\ Q = W_{Q}^{{}} .X_{att - input}^{{}} \hfill \\ \end{gathered} \right. \to \alpha = SoftMax(QK^{T} ) $$

5 $$ V = W_{V}^{{}} .X_{att - input} \to^{{}} X_{att - output} = \alpha V $$

Where $\alpha$ contains the attention weights,$W_{k}^{{}}$, $W_{Q}^{{}}$, and $W_{V}^{{}}$are defined through the training process and the SoftMax function ensures that the sum of attention weights is equal to 1. It is important to note that in our model, each component of the output from the attention block ($X_{att - output}$) contains information from all the features of the input signal ($X_{att - input}$), thanks to the use of attention weights. This cannot be achieved using simple Conv3D or SE-Res blocks. The incorporation of this characteristic in the attention block plays a crucial role in enhancing the performance of our proposed MASERes model.

### Ethics declarations

This research is a prospective descriptive study that was approved by the Mazandaran University of Medical Science Ethics Committee (IR.MAZUMS.REC. 1402.14368) and carried out in accordance with the ethical guidelines of the Helsinki Declaration Principles.

## Results

### Assessment metrics

To present the performance evaluation of models in both slide-level analysis and patient-level analysis, standard metrics commonly used in classification tasks are utilized. These metrics include accuracy, precision, recall or sensitivity, specificity, and f1-score. The equations below demonstrate how these metrics are calculated, and a higher value for each metric indicates better performance of the model. Additionally, the area under the receiver operating characteristic curve (AUC) is reported for each model. AUC provides a graphical representation of the model's ability to distinguish between positive and negative classes by analyzing various classification thresholds. A value of 1 for AUC indicates a perfect classifier, while a value of 0.5 indicates a random classifier.

In the following equations, TP refers to true positive (normal samples correctly classified by the model), TN refers to true negative (COVID-19 samples correctly classified by the model), FP refers to false positive (COVID-19 samples incorrectly classified by the model as normal), and FN refers to false negative (normal samples incorrectly classified by the model as COVID-19). Note that in our study, COVID-19 samples are considered as negative samples while normal samples are positive.

6 $$Accuracy= \frac{TP+TN}{TP+TN+FP+FN}$$

7 $$Precision= \frac{TP}{TP+FP}$$

8 $$Recall or Sensitivity= \frac{TP}{TP+FN}$$

9 $$Specificity= \frac{TN}{TN+FP}$$

10 $$F1-Score= \frac{2 \times Precision \times Recall}{Precision+ Recall}$$

### Slide-level results

**Figure 4:**
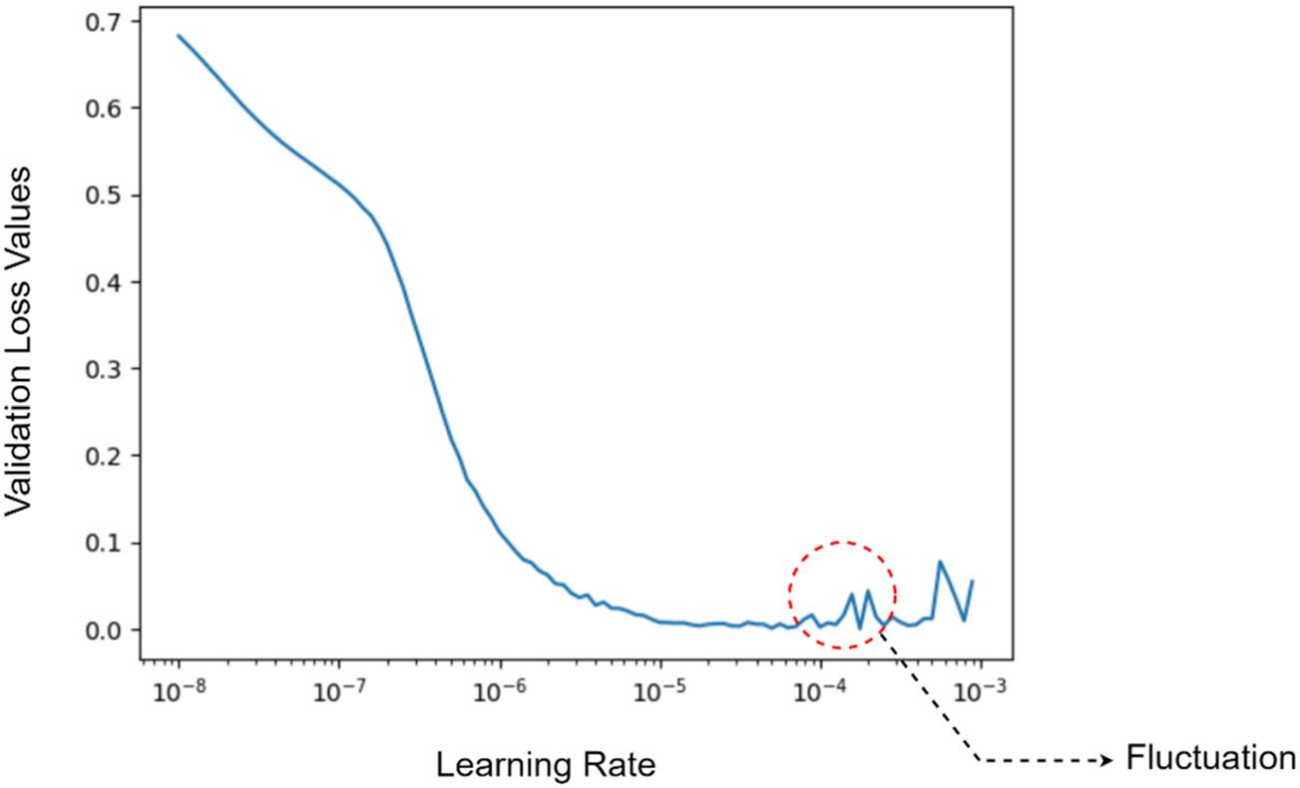
Learning rate scheduler technique used to determinet the proper learning rate value for ResNet50V2.

With the objective of performing a binary classification task, we employed Binary Cross Entropy as the loss function. Several optimization algorithms were evaluated in this study, which encompassed Adam^56^ with a learning rate of 0.001, RMSProp^57^ with a learning rate of 0.001, and SGD with a learning rate of 0.001 and a momentum value of 0.9. After careful examination, it was determined that Adam outperformed the other optimizers, and consequently, it was selected as the preferred optimization algorithm for this task. To determine an optimal value for the learning rate, we implemented learning rate scheduler method, as recommended in the reference^58^. In this technique, the network is trained on the dataset in 100 epochs with incremental learning rate values starts from 10^–8^ to 10^–3^. For each model, a plot has been generated to analyze the relationship between loss values on the validation set and learning rate values which represented the impact of varying learning rates on the loss function during model training. Initially, the values exhibit a gradual decline, but it is noted that after a certain point, fluctuations begin. Therefore, a specific point was identified before the onset of the observed fluctuations, which was specifically targeted for further training and optimization to prevent any potential instability. Figure 4 outlines this methodology to identify an optimal learning rate value for ResNet50V2. The graph illustrates that the loss values exhibit fluctuation after reaching a value of approximately 10^–4^. Therefore, values such as 7 × 10^–5^ or 8 × 10^–5^ have the potential to serve well as the learning rate. Nonetheless, in consideration of computational efficiency, 10^–5^ was selected as the optimal learning rate to train this model.

In the process of selecting an appropriate batch size for model training, we conducted an evaluation using various batch sizes, specifically [16, 32, 64, 128]. After thorough analysis, it was determined that a batch size of 32 yielded the most optimal performance. Consequently, a batch size of 32 was adopted for the training process. The number of epochs, initially set to 50, is controlled using the early stopping callback, which stops the training process if the loss function on the validation data does not improve for 10 epochs. TensorFlow^59^ is used to implement the models, and they are trained on the Kaggle environment using a GPU100 accelerator. Table 3 provides a summary of the training process used for the slide-level analysis.

**Table 3:** Details regarding the training process of each slide-level model.

Model	Input image size	learning rate	Num of epochs
VGG19^38^	(224 × 224)	10^–5^	50
InceptionV3^39^	(299 × 299)	10^–5^	50
ResNet50V2^40^	(224 × 224)	10^–5^	33
Xception^41^	(299 × 299)	10^–6^	50
MobileNetV2^42^	(224 × 224)	10^–6^	50
DenseNet169^43^	(224 × 224)	10^–5^	50

Various models were utilized as the pre-trained model for the transfer learning technique used in this study and each model had a specific input shape size. For instance, some of the models were suitable for images with a size of 224 × 224, while others could only work with images of size 299 × 299. To exploit the initial weight values of these models obtained through training on the ImageNet Large Scale Visual Recognition Challenge (ILSVRC)^44^ dataset, images are resized to a suitable size for each model respectively and thereby input size varies among different models. After the resizing of the input images, all pixels of them were rescaled to a range from 0 to 1. To implement data augmentation, images were randomly rotated from 0 up to 40°, randomly shifted from 0 to 20% in width and height, randomly zoomed from 0 to 20%, and randomly flipped horizontally.

**Figure 5:**
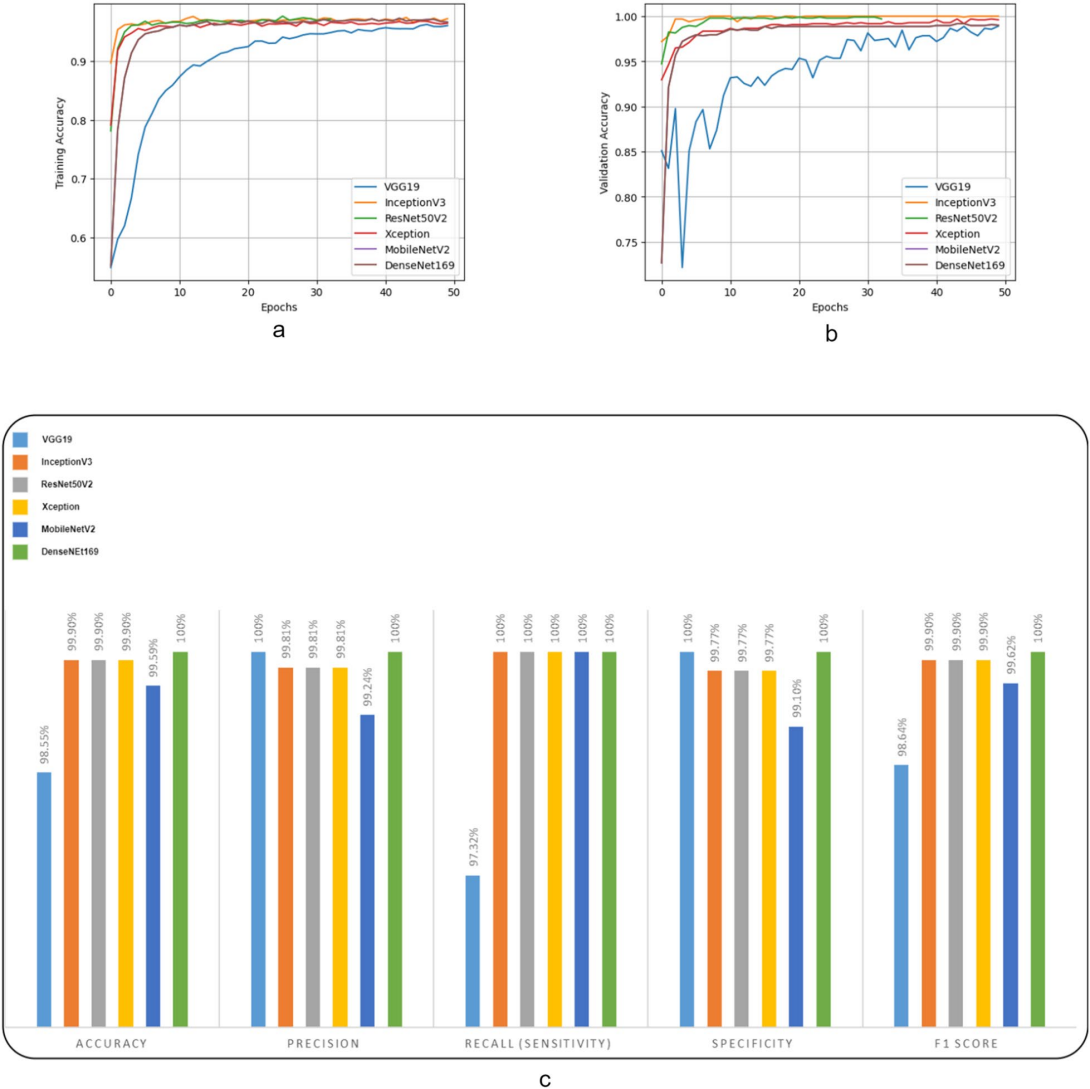
The accuaracy of various slide-level models during the training process on both the training set (**a**) and validation set (**b**). The performance of various slide-level models across different evaluation metrics on the test set (**c**).

The performance of various proposed models with different pre-trained networks is displayed in the Fig. 5c. With the exception of the model using VGG19 as the pre-trained architecture, all models achieved an accuracy of over 99%. Moreover, the sensitivity values of the models indicate that all models, except for VGG19, can accurately detect normal slides. This means that if these models classify a given slide as normal, it is highly likely to be correct, and no normal slide is classified as COVID-19 with these models (as normal slides are considered as positive samples in this study). However, the specificity metric values of the models demonstrate a slight weakness in detecting COVID-19 slides. Thus, if a slide is classified as COVID-19 by these models, further evaluations may be necessary. Overall, the transfer learning model based on DenseNet169 achieved a 100% performance in all metrics and is a suitable model for slide-level analysis. Furthermore, it should be noted that the area under the curve (AUC) value for all the slide-level models was 1. This indicates that the models had a perfect discriminatory ability in distinguishing between COVID-19 positive and negative cases at the slide level.

Figure 5a,b illustrates the accuracy achieved by various models during the training process on both the training and validation sets. The results demonstrate that all models, except for the one based on VGG19, achieved high accuracy with only a few epochs. In contrast, the VGG19-based model had a slow convergence to high accuracy values in both the training and validation sets. Additionally, the final accuracy of the VGG19-based model was lower compared to the other models.

### Patient-level results

For the patient-level analysis, binary Cross Entropy was employed as the loss function given the binary classification task. Furthermore, in a manner analogous to the slide-level analysis, we employed the optimization algorithms Adam^56^, SGD, and RMSProp^57^ during the training process for patient-level analysis. After comprehensive assessment, it was ascertained that Adam exhibited superior performance, thus leading to its selection as the optimizer for the patient-level analysis as well. The optimal learning rate was determined using the learning rate scheduler method recommended in^58^, which is described in detail in section "Slide-level results". The models were trained for 50 epochs, during which various minibatch sizes, namely^2,4,8^, were examined to identify the optimal choice. After careful consideration, a minibatch size of 2 was deemed to be the most suitable and was consequently adopted for the training process. TensorFlow^59^ was used to implement the models, which were trained on the Kaggle environment with a GPU100 accelerator.

To establish a benchmark for comparison with the final results of MASERes, two other models, 3D-AlexNet and VoxCNN, were implemented on the dataset. These models are specifically designed for 3D image data and thereby capable of performing accurately in CT volume classification.

3D-AlexNet is the 3D version of the AlexNet^60^, the winner of the ImageNet Large Scale Visual Recognition Competition in 2012 (ILSVRC2012). AlexNet is specifically designed for image classification and as a result is not suitable for the 3D inputs. To make it possible to implement this model on the CT volumes, we substituted every layer of the original model with its corresponding 3D counterpart. On the other hand, VoxCNN were introduced in^61^ for the classification of MRI 3D images and is created by replacing the layers in VGG^38^ with their corresponding 3D versions. Table 4 outlines the specifics of the training process utilized in the patient-level analysis.

**Table 4:** Details regarding the training process of each patient-level model.

Model	Input image size	learning rate	Num of epochs
3D-AlexNet	(128 × 128 × 128)	5 × 10^–7^	50
VoxCNN^61^	(128 × 128 × 128)	7 × 10^–6^	50
MASERes	(128 × 128 × 128)	10^–6^	50

To enhance the robustness and diversity of the patient-level models, we employed data augmentation techniques. Specifically, we randomly rotated the volumes in one of the following degrees: − 20, − 10, − 5, 0, 5, 10, or 20. By incorporating these variations, we aimed to increase the variability of the dataset, thereby improving the generalizability and accuracy of our results.

**Figure 6:**
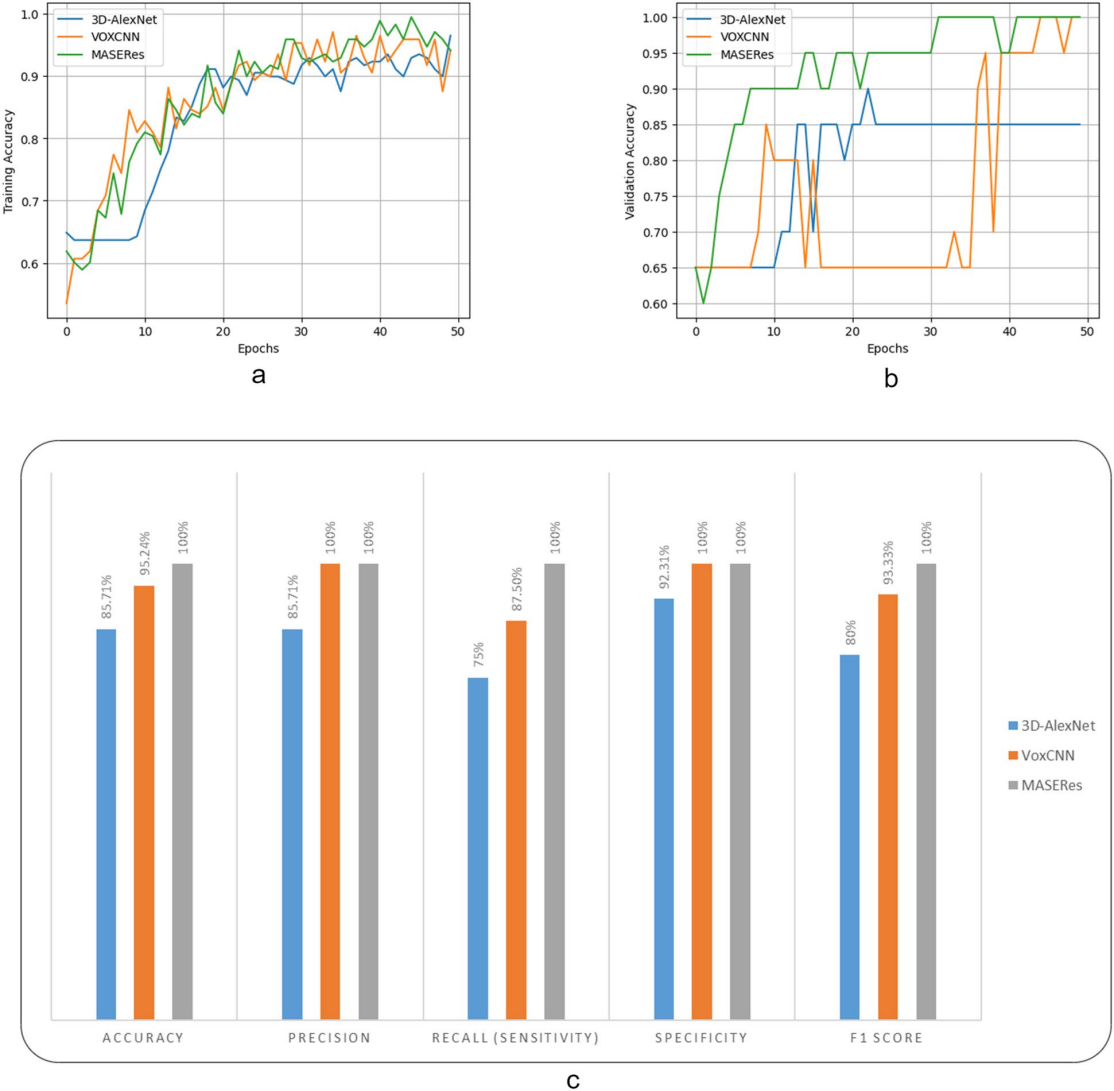
The accuaracy of various patient-level models during the training process on both the training set (**a**) and validation set (**b**).The performance of various patient-level models across different evaluation metrics on the test set (**c**).

Figure 6c displays the performance of various models for patient-level analysis on CT scan volumes. The results indicate that the proposed MASERes model outperforms all benchmark deep models and achieves a 100% performance on all metrics. This finding demonstrates the efficacy of the proposed model over the benchmark models. The AUC value for the 3D-AlexNet model was 0.9712, whereas it was 1 for both the VoxCNN and MASERes models.

Furthermore, Fig. 6a,b illustrates the changes in accuracy on the training and validation sets during the training process. The results show that the accuracy convergence of all models is similar during the training process. However, on the validation set, the proposed MASERes model converges to high accuracy with fewer epochs compared to the benchmark models.

In order to assess the robustness of MASERes, we conducted an analysis that involved the calculation of both z-scores and kappa scores. In Table 5, we present the *p*-values derived from a z-test, which were helpful in comparing the performance of MASERes against two benchmark models across various metrics. Our findings indicate that there is a significant difference in the performance of MASERes when compared to 3D-AlexNet across all metrics, with the exception of specificity, where the *p*-value exceeds 0.05. Furthermore, when contrasting MASERes with VoxCNN, a significant difference in sensitivity is observed. In addition, both 3D-AlexNet and VoxCNN exhibit agreement with MASERes, as indicated by their respective Cohen’s kappa scores of 0.5570 and 0.8966. The highest agreement (kappa = 0.8966) was demonstrated between MASERes and VoxCNN which means that the combination of these two models has the most coverage of patient diagnosis that in turn strengthen the power of studies in this field.

**Table 5:** The p-values obtained via z-test and performance differential of MASERes in various metrics compared to benchmark models.

Metrics	MASERes versus 3D-AlexNet	MASERes versus VoxCNN
Accuracy	0.031*	0.153
Precision	0.031*	0.500
Sensitivity	0.004*	0.042*
Specificity	0.093	0.500
F1 Score	0.011*	0.110

* p-values < 0.05.

#### MASERes on COVID-CTset

In order to evaluate the efficacy of the MASERes model in the present study, an assessment was conducted using the COVID-CTset dataset^26^. The COVID-CTset is a publicly available repository comprising CT scan images, which encompass 95 cases of COVID-19 patients and 282 instances of normal individuals. These CT scan images were acquired at Negin Radiology, situated in Sari, Iran, during the period spanning from March 5th to April 23rd, 2020. Notably, all CT scan images within this dataset possess dimensions of 512 × 512 pixels and are encoded in grayscale TIFF format.

COVID-CTset dataset serves solely as an evaluation benchmark for the proposed model architecture, without any hyperparameter optimization performed on it. Consequently, this dataset is partitioned into two distinct subsets: training and validation, with an allocation ratio of 80% for training and 20% for validation. Specifically, the training subset encompasses CT scan images of 301 patients (76 COVID-19 and 225 normal) while the validation subset comprises 76 samples (19 COVID-19 and 57 normal cases). Given the grayscale nature of the data within the COVID-CTset, characterized by a single channel, an adaptation was made to the input layer of the MASERes model. The input layer dimension was adjusted from (128 × 128 × 128 × 3) to (128 × 128 × 128 × 1) to align with this characteristic. Beyond this minor modification, all hyperparameters previously established for training the MASERes model on the 3D-COVID-MAH-CT dataset were retained for its application to the COVID-CTset. These consistent hyperparameters encompass a learning rate of 10^–6^, a mini-batch size of 2, and the utilization of the Adam optimizer^56^.

**Figure 7:**
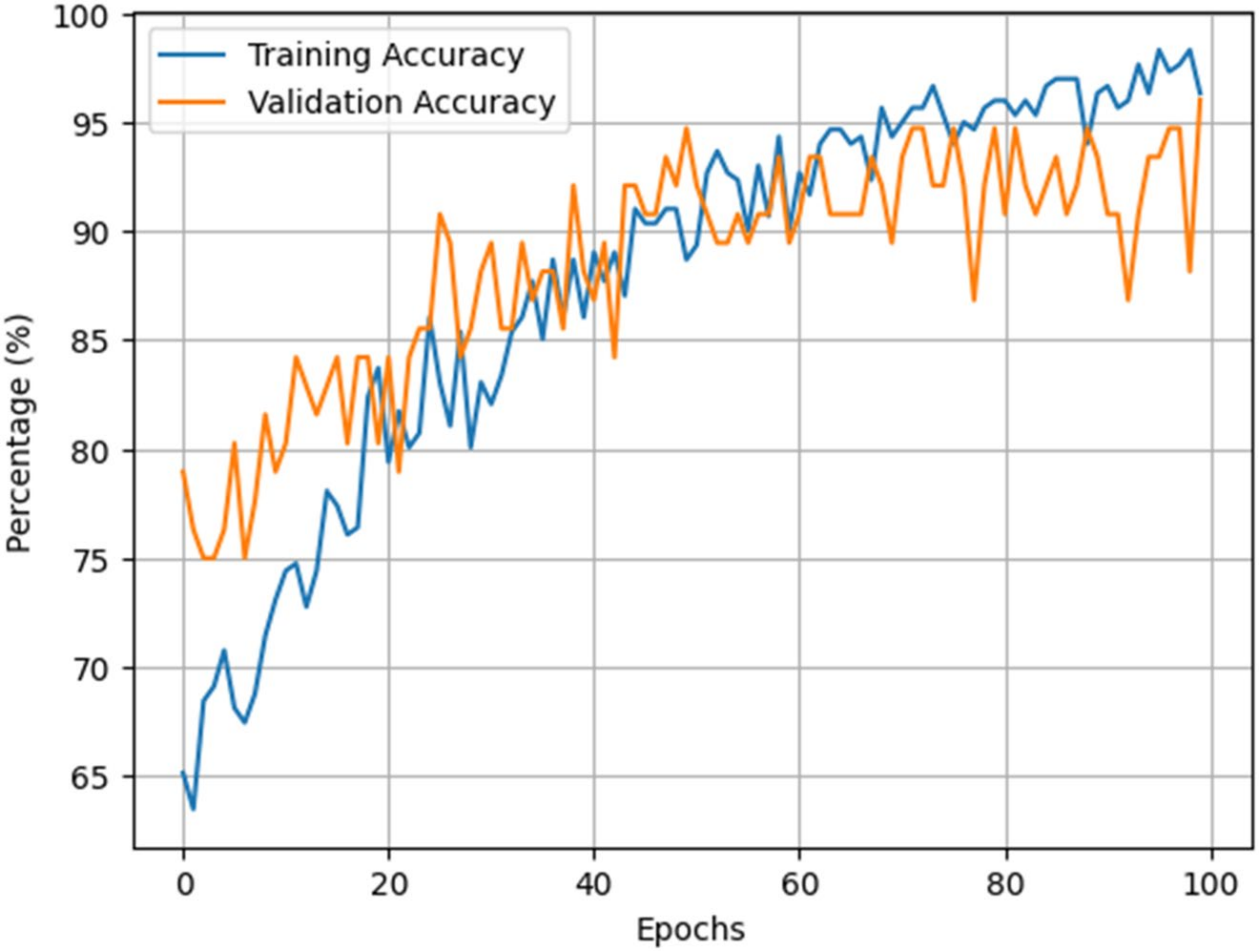
Performance graph of the MASERes model during training phase on COVID-CTset.

Figure 7 illustrates the progression of accuracy throughout the training phase of the MASERes model when applied to the COVID-CTset. Furthermore, Table 6 provides a comprehensive overview of the model's performance, presenting various metrics relating to the validation subset. It is evident from Table 6 that the accuracy achieved by the MASERes model on the COVID-CTset is an impressive value of 96.05%. This achievement is notably comparable to the original algorithm's performance proposed in^26^, which was designed for the identification of COVID-19 patients within COVID-CTset and yielded an accuracy of 95.58%. This difference underscores that the structure of MASERes proposed in this study can accurately identify patients in the covid-CTset dataset.

**Table 6:** Performance of the MASERes model on COVID-CTset.

Accuracy	precision	Sensitivity:	Specificity:	f1-score:
96.05%	95.0%	100.0%	84.21%	97.44%

#### Ablation study

Ablation study in the field of machine learning involves systematically removing or disabling specific components or elements of a model, such as layers, features, or parameters, to understand their individual contributions to the model's performance. The primary goal of an ablation study is to gain insights into the model's behavior, identify which components are essential, and assess the impact of each component on the model's overall performance.

We conducted an ablation analysis to assess the importance of different components within the MASERes model. To achieve this, we opted to reduce the influence of each component rather than completely removing it. Full removal of a component would disrupt the information flow from preceding layers to subsequent layers, which is not desirable for our analysis. Therefore, we chose a more nuanced approach by eliminating only 25% of the parameters within the trainable layers of each component as suggested in^62^. This was accomplished by manually setting the weights (w) and bias values to zero. It's worth noting that the actual number of neurons and filters in each layer may vary, resulting in different quantities of filters and neurons being removed at different layers.

**Figure 8:**
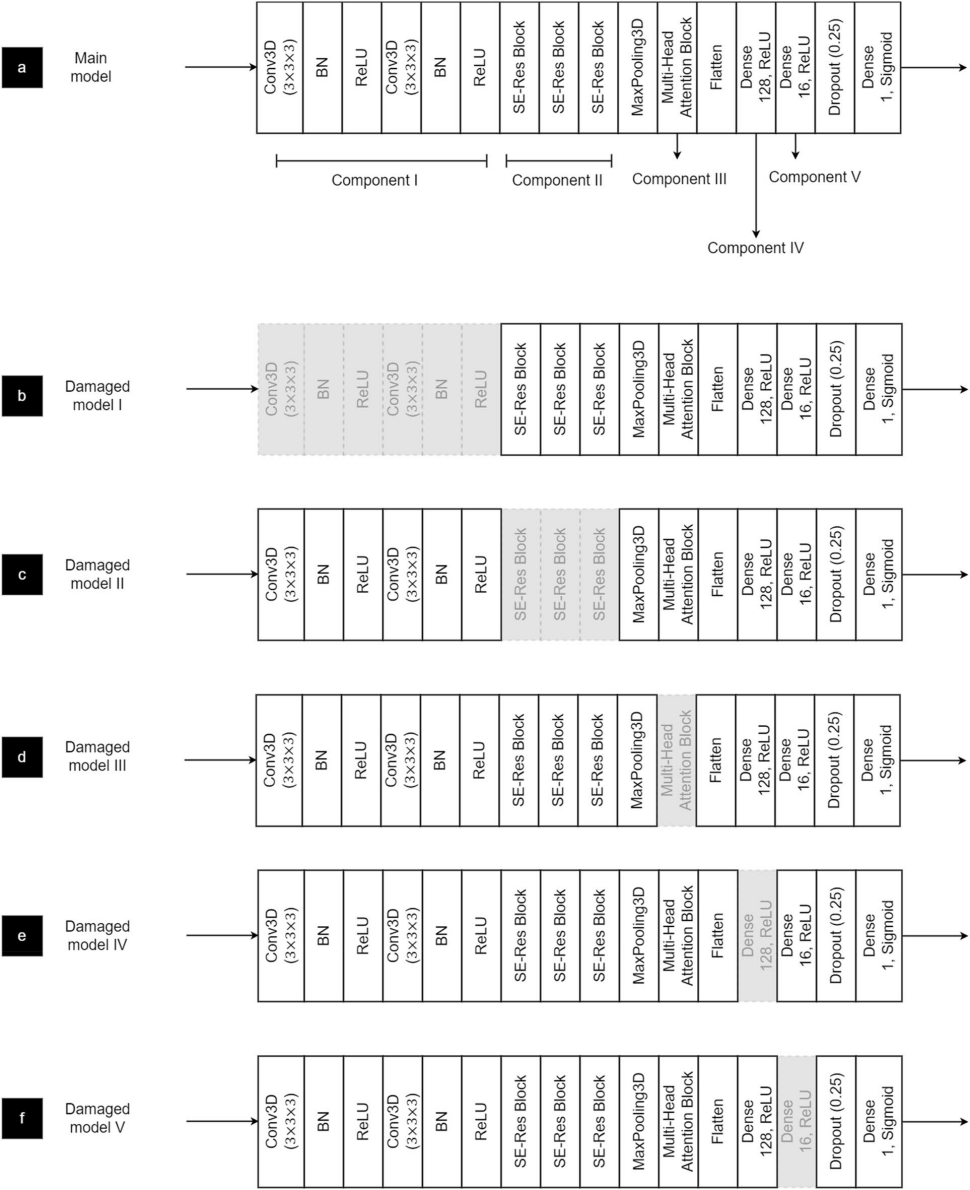
The structures of the main model (**a**) and the damaged models after ablating different components. Ablation of component I, II, III, IV, and V resulted in the damaged model I (**b**), damaged model II (**c**), damaged model III (**d**), damaged model IV (**e**), and damaged model V (**f**) respectively.

Figure 8a provides an overview of the main model's architecture, highlighting its components. Component I, comprising two identical blocks, serves as the foundational layers responsible for extracting low-level features. We removed 4 filters out of the 16 filters in each block and created a modified model known as damaged model I, illustrated in Fig. 8b.

In Component II, there are three identical SE-Res blocks that share the same structure. These blocks analyze the features from Component I to identify higher-level features and important channels using the squeeze-and-excitation technique. To create damaged model II (see Fig. 8c), we simply removed 25% of the convolutional filters and fully-connected layer neurons from this component in the original model.

Component III is a multi-head attention block designed to discern crucial spatial features exclusively comprising fully connected layers. We removed 25% of the neurons from each layer within this component of the original model which resulted in damaged model III as depicted in Fig. 8d.

Following the extraction of crucial features from the input data through distinct convolutional layers within the MASERes structure, these features are subsequently inputted into two fully connected layers comprising 128 and 16 neurons, respectively, for the ultimate classification task. Firstly, we removed 32 neurons from the initial fully connected layer, resulting in the creation of Damaged Model IV (as depicted in Fig. 8e). Subsequently, we proceeded to extract 4 neurons from the second fully connected layer, thereby yielding Damaged Model V (visible in Fig. 8f).

After reducing the effect of distinct components, an assessment of the respective damaged models has been conducted using the test data. The accuracy of each of these damaged models are comprehensively documented in Table 7. An in-depth analysis of this metric and its comparison against the original model's performance, facilitates a comprehensive understanding of the impact associated with the removal of individual components.

**Table 7:** the performance of various damaged models during ablation study on classifying COVID-19 and normal samples.

Model	Accuracy on detecting COVID-19 cases (%)	Accuracy on detecting normal cases (%)	Total accuracy (%)
Main model	100	100	100
Damaged model I	15.38	100	47.62
Damaged model II	100	62.5	85.71
Damaged model III	100	0	61.90
Damaged model IV	100	87.5	95.24
Damaged model V	100	100	100

Based on the information presented in the Table 7, Component I plays a crucial role in influencing the model's performance in identifying COVID-19 samples. A reduction in the influence of this component results in a substantial decline in the model's ability to accurately classify COVID-19 data. Conversely, the other components contribute significantly to the main model's capability to identify normal samples. Notably, Component IV, multi-head attention, exhibits a particularly pronounced impact. When the influence of Component IV is diminished, "MASERes," experiences a complete inability to correctly identify any normal samples. Furthermore, the findings from Damaged Model V reveal that the final features are distributed within the 16 neurons of the final layer in such a manner that the removal of a portion of these neurons does not exert a discernible impact on the overall performance of the original model.

## Discussion

Our research findings affirm the significant utility of the COVID-MAH-CT dataset in the development of deep learning methodologies. This dataset encompasses a diverse array of COVID-19 manifestations observed across four distinct waves, thus validating its comprehensiveness. Notably, our analysis demonstrates that six distinct transfer learning models achieve an exceptional accuracy rate of 99% when applied to slide-level analysis of the COVID-MAH-CT dataset. In conjunction with this, we introduce a novel deep learning model, MASERes, designed for patient-level analysis. MASERes exhibits a high degree of accuracy in identifying COVID-19 patients based on 3D CT volumes. The implementation of MASERes carries several compelling advantages. Firstly, the early detection of COVID-19 through MASERes translates into a reduction in patient mortality rates. The automated diagnosis enabled by this model ensures that individuals are identified at the onset of the disease, consequently lowering the overall mortality risk. Secondly, MASERes enhances the efficacy of the treatment process. Its precise identification of COVID-19 from 3D CT volumes, as verified on different datasets, optimizes the treatment routine, leading to improved patient outcomes. Lastly, MASERes plays a pivotal role in improving the stress on healthcare infrastructure, particularly concerning the occupancy rates of hospital beds, which is of top importance during pandemic and endemic situations. By swiftly and accurately identifying COVID-19 cases, MASERes aids in efficiently allocating resources and managing the patient load, thereby contributing to the overall preparation and resilience of healthcare systems in the face of health crises.

Several research studies have employed deep learning techniques for slide-level and patient-level analyses. An issue that became evident in the early stages of utilizing deep learning for COVID-19 detection from CT scans was the lack of transparency in sharing the datasets used for model training^19,20^. This limitation is understandable given the novelty of COVID-19 and the initial absence of suitable datasets. Nevertheless, it is crucial to emphasize that the publication of COVID-19 datasets carries significant benefits. Not only does it permit the replication of research findings, but it also plays a pivotal role in fostering the development of other novel deep learning models for the precise detection of COVID-19.

Numerous additional studies have dedicated their efforts to the development of deep learning models for slide-level analysis^21–25^. These models are primarily designed to assess individual slices within a series of CT scans obtained from a single patient, discerning whether a given slice exhibits signs of COVID-19 infection. It is worth noting that the slices used for training these models often pertain to different patients, potentially originating from varying angles of image capture. Consequently, the features learned from one slice may not necessarily be transferable or applicable to another slice acquired from a different angle. While these studies have successfully developed models capable of detecting COVID-19 in individual CT slices, it is essential to underscore their inherent limitation—they do not possess the capability to classify patients as either COVID-19-infected or uninfected.

To achieve patient-level COVID-19 analysis, numerous research studies have been conducted, and a selection of the state-of-the-art investigations is briefly presented in Table 8. These studies frequently employ 2D deep learning models to individually assess each slice within a series of CT scans. Subsequently, the collective findings from these slide-level analyses are combined through decision or voting methodologies to arrive at a conclusive patient classification. Nevertheless, it is imperative to acknowledge that this method introduces certain challenges and limitations.

**Table 8:** State of the art studies on detecting COVID-19 patients using CT scans.

Study	Method	Dataset	Performance	Limitations
Wu et al.^63^	A CNN based on ResNet50	Self-collected dataset*	Accuracy: 76%	*Not end-to-end *Discarding slices (only keeping one slice for each view)
Rahimzadeh et al.^26^	Combination of ResNet50V2^40^ and Feature pyramid network (FPN)^64^	COVID-CTset**	Accuracy: 95.58%	*Not end-to-end *Discarding slices
Serte et al.^27^	ResNet50^46^	Mosmed-1110^65^ and CCAP^66^	AUC: 96%	*Not end-to-end *Discarding slices
Kollias et al.^28^	Combination of CNN (extracting features) and RNN models (calculating probability)	COV19-CT-DB***	F1-score: 67%	*Not end-to-end *Every slide of a patient is not individually labeled
Fallahpoor et al.^67^	3D ResNet50	Iranmehr**** Mosmed-1110^65^	AUC: 92%	*Computationally expensive *Losing information by cropping CT slices

* 368 COVID-19 patients and 127 patients with other pneumonia collected from three hospitals in China.

** a dataset collected by the authors from Negin Radiology Medical Center in Sari, Iran which contained 48,260 CT scan images from 282 normal persons and 15,589 images from 95 patients with COVID-19 infections.

*** a dataset containing about 5000 3D CT scans belong to more than 3000 patients and subjects.

**** a dataset containing 1110 3D CT scans collected from Iranmehr hospital, Tehran, Iran.

Firstly, these models require the meticulous labeling of each individual CT slice within a patient's scan series, an essential component of supervised learning. This labeling process is labor-intensive, rendering it both time-consuming and economically demanding.

Secondly, in an attempt to reduce computational costs, many of these investigations have sought to narrow the focus to a subset of CT slices that comprehensively depict the patient's lung, rather than utilizing the entire array of slices in the scan series. Various techniques have been employed to select these representative slices from the CT scans, with varying strategies being employed in different studies. For instance, Rahimzadeh et al.^26^ utilized an approach that involved evaluating pixel values within a particular central section of each slice. In contrast, Kollias et al.^28^ opted for a different method, selecting the median slice along with its preceding and succeeding slices for every patient. However, this selective approach introduces challenges during both the training and testing phases. In the training phase, the model's efficacy may be compromised, as the excluded slices may contain valuable information pertinent to COVID-19 detection. It is plausible that the manifestations of COVID-19 exclusively manifest in slices where the patient's lung is not entirely captured (i.e., the eliminated slices) and remain absent from the selected slices, potentially undermining the deep learning model's performance. Moreover, in the testing phase, where the model is tasked with making predictions for individual patients, the initial step involves selecting the desired slices, which may not exhibit COVID-19 manifestations. Consequently, the model is susceptible to erroneously classifying a COVID-19-infected patient as a healthy individual, a scenario that could exacerbate the chain of COVID-19 transmission. Such a situation presents a serious concern, particularly during endemic and pandemic conditions, as it places significant strain on quarantine policies and public health efforts.

Finally, it is notable that these studies have incorporated a range of preprocessing and postprocessing steps, which essentially transform their deep learning models into multi-step techniques rather than end-to-end models. During the testing phase and the classification of new samples, a multi-step model entails a considerable time investment. Conversely, an end-to-end model, like MASERes, devoid of the necessity for additional processing steps, can efficiently receive the CT slices and promptly yield predictions. This attribute assumes principal significance in circumstances demanding swift detection, notably during critical situations like pandemics and endemic conditions.

Fallahpoor et al.^67^ conducted a study aimed at assessing the generalization capabilities of deep learning models trained on 3D CT volumes of COVID-19 patients. They employed a methodology analogous to our own, involving the utilization of 3D convolutional layers to process the aforementioned CT volumes. They also interpolated 3D CT volumes to have the same number of slices for all patients, similar to our study. However, instead of directly inputting these interpolated 3D CT volumes into their model, Fallahpoor et al. introduced two supplementary preprocessing stages intended to selectively retain the most important parts of each slice. They initially cropped all slices to diminish their dimensions and subsequently employed a segmentation model to generate specific masks for individual slices, thereby isolating the lung region. These additional preprocessing steps may be conducive to a suboptimal performance of their proposed technique.

Cropping each slice has the potential to result in the inadvertent loss of vital information contained within the slices. Furthermore, the segmentation process, being intrinsically prone to errors, has the potential to introduce cumulative inaccuracies, which, in turn, could compromise the overall performance of the model. It is also noteworthy that the segmentation component is likely to introduce a substantial computational overhead, both during the training and testing phases. This is primarily attributable to the necessity of employing different segmentation models for varying types of CT slice formats, as explained in their study.

Di Napoli et al.^68^ also conducted a study using 3D convolutional layers to analyze 3D CT volumes of COVID-19 patients. However, their primary goal differs from ours, as their study is centered on predicting outcomes such as mortality, intubation, and ICU admission among COVID-19 patients, rather than identifying COVID-19 cases.

Furthermore, there is another pertinent study conducted by Kermi et al.^69^ which similarly leverages 3D convolutional layers for the examination of COVID-19 3D CT volumes. Nevertheless, the core objective of their study differs significantly from ours, as their primary aim is not the detection of COVID-19 patients but rather the segmentation of COVID-19 lesions within 3D CT volumes associated with COVID-19 cases.

### Limitations

One of the limitations of this study is the absence of CT samples from patients with pulmonary conditions other than normal or COVID-19-infected subjects, such as pneumonia or tuberculosis. Another limitation of the present study relates to the size of the dataset, particularly in patient-level analysis. Although we addressed this issue in slide-level analysis by utilizing transfer learning, this challenge still exists in patient-level analysis.

## Conclusion

In this study, we proposed and evaluated six different end-to-end transfer learning models that achieved high accuracy in slide-level analysis for detecting COVID-19 in CT scan images. Moreover, we developed a MASERes, a novel deep model that can accurately analyze 3D CT volumes for patient-level analysis. Both of these models are end-to-end, eliminating the need for expert analysis of CT scan images and reducing the additional costs associated with COVID-19 diagnosis. Additionally, we introduced COVID-MAH-CT, a publicly available dataset of COVID-19 CT images and 3D CT volumes. This dataset can be valuable for further research using deep and machine learning techniques to improve COVID-19 detection in CT scans.

## Data Availability

We have made our data available for public use in this address: (https://github.com/alrzsdgh/COVID). The dataset is available in two version: 2D-COVID-MAH-CT and 3D-COVID-MAH-CT. The former one contains COVID-19 infected CT slides while the latter one contains 3D CT scan volumes. We hope that this dataset will be utilized for improving COVID-19 monitoring and detection in the coming researches.

## Code availability

We would like to clarify that all codes employed for data analysis, including training, validation, testing, and the trained networks for both slide-level and patient-level analysis, have been made publicly available at (https://github.com/alrzsdgh/COVID).
